# One-Step Solvothermal Synthesis of Fe_3_O_4_ Acicular Aggregates Induced by Reaction Medium and Urea for Photocatalytic Degradation of Azo Dyes

**DOI:** 10.3390/nano15050341

**Published:** 2025-02-22

**Authors:** Yaohui Xu, Yuting Li, Quanhui Hou, Liangjuan Gao, Zhao Ding

**Affiliations:** 1 Laboratory for Functional Materials, School of New Energy Materials and Chemistry, Leshan Normal University, Leshan 614000, China; xyh1986@lsnu.edu.cn; 2 Leshan West Silicon Materials Photovoltaic and New Energy Industry Technology Research Institute, Leshan 614000, China; 3 College of Materials Science and Engineering, National Engineering Research Center for Magnesium Alloys, Chongqing University, Chongqing 400044, China; lyt@wust.edu.cn; 4 School of Automotive Engineering, Yancheng Institute of Technology, Yancheng 224051, China; hqhdyx66@ycit.edu.cn; 5 College of Materials Science and Engineering, Sichuan University, Chengdu 610065, China; lgao87@scu.edu.cn

**Keywords:** Fe_3_O_4_, solvothermal, photocatalyst, UV irradiation, degradation, azo dye

## Abstract

Based on the magnetic sensitivity of Fe_3_O_4_ in various fields, we aimed to propose a one-step solvothermal process for the synthesis of single-phase Fe_3_O_4_ induced by the reaction medium and urea, avoiding high-temperature reduction in H_2_ or N_2_ atmospheres. Feasibility was tested with purified water (H_2_O), methyl alcohol (MA), ethyl alcohol (EA), and ethylene glycol (EG) as reaction media. The findings indicated that the solvothermal reaction system utilizing EA was more effective for the synthesis of cubic magnetic Fe_3_O_4_. Optimal conditions for synthesizing pure Fe_3_O_4_ were obtained by optimizing the urea amount and solvothermal reaction parameters. The optimal formulation consisted of 10 mmol of FeCl_3_, 80 mmol of urea, and 60 mL of EA subjected to a solvothermal process at 200 °C for 12 h. The resulting Fe_3_O_4_ (magnetite, cubic) exhibited commendable crystallization with a morphology of acicular aggregates and displayed excellent magnetic sensitivity properties with a magnetization of 92.2 emu/g at 15,000 Oe. The photocatalytic degradation behaviors of the resulting Fe_3_O_4_ to Methyl Orange, Orange G, and Acid Red 37 azo dyes and the repeated degradation performance of Methyl Orange dye were investigated. Nearly complete degradation of Methyl Orange dye occurred after 2.0 h of photocatalytic reaction, while Orange G and Acid Red 37 dyes achieved similar results after 3.5 and 4.5 h, respectively. The exploration strategy in this work for synthesizing magnetic Fe_3_O_4_ can be applied to design and fabricate other metal oxides or composites, potentially resulting in novel discoveries in morphology or performance.

## 1. Introduction

Azo dyes, distinguished by at least one nitrogen-to-nitrogen double bond (−N=N−), comprise the most numerous varieties of synthetic dyes, including acid, mordant, reactive, cationic, neutral, and disperse dyes, among others, accounting for 80% of organic dye products and exhibiting complete chromatography. Most azo dyes are formed through the combination of aromatic amines with phenols or active methylene compounds following heavy ammoniation. These dyes have a strong capacity to absorb sunlight and prevent sunlight from entering the water, thereby lowering dissolved oxygen levels and reducing photosynthesis in aquatic plants, which has a particularly devastating impact on the aquatic ecosystem, causing catastrophic consequences for aquatic organisms. Additionally, numerous azo dyes have been found to possess carcinogenic properties or exhibit toxicity in the human body [1]. Therefore, it is imperative to thoroughly decontaminate water prior to its reuse [2,3]. However, as a result of the relatively stable chemical properties of azo dyes, the composition of wastewater containing them is intricate, presenting a formidable challenge in the treatment of these organic effluents. Photocatalysis stands out as a highly effective approach for the remediation of water polluted by dyes, particularly among other advanced oxidation processes (AOPs) [4,5]. This is due to its capability to operate under ambient conditions of temperature and pressure without generating any secondary byproducts [6].

A variety of materials have been identified as highly efficient photocatalysts, such as titanium dioxide (TiO_2_) [7], zinc oxide (ZnO) [8], (MnO_2_) [9], zinc sulfide (ZnS) [10], cadmium sulfide (CdS) [11], Cu_2_ZnSnS_4_ (CZTS) [12], tungsten trioxide (WO_3_) [13], strontium titanate (SrTiO_3_) [14], lithium niobate (LiNbO_3_) [15], and alpha-iron oxide (*α*−Fe_2_O_3_) [16], bismuth oxychloride (BiOCl) [17], bismuthyl bromide (BiOBr) [18] and their compounds or solid solutions [19,20,21,22,23]. Further, in addition to demonstrating excellent catalytic activity and high stability, the ability to be recycled is another crucial property for catalysts. It is widely recognized that traditional separation techniques, such as filtration and centrifugation, are often time-consuming and inefficient, making them unsuitable for the recycling of catalysts. In contrast, magnetic separation offers a distinct advantage as it provides a convenient method for removing and recycling magnetic particles and/or composites through the application of an external magnetic field. This approach not only reduces losses of the catalyst during the recycling process but also enhances the durability of the catalyst overall.

The choice of magnetite (Fe_3_O_4_) nanomaterials as catalysts can offer the additional advantage of facilitating easy magnetic separation of the catalyst from water [24,25]. Most importantly, the selection of Fe_3_O_4_ enables the utilization of a photo-Fenton-type reaction, providing a non-toxic, cost-effective, and environmentally friendly method for decontaminating industrial-grade dyes. In recent studies, the primary method for preparing Fe_3_O_4_ involved the hydrogen (H_2_) reduction of *α*−Fe_2_O_3_ [26,27]. This process utilized *α*−FeO(OH) or *α*−Fe_2_O_3_ as the raw materials, where the air was initially completely replaced by high-purity nitrogen (N_2_). Subsequently, the system was slowly introduced into water-saturated hydrogen through a wash cylinder while simultaneously raising the temperature to 300~400 °C and maintaining it for 1~3 h. Jiao [28] synthesized mesoporous Fe_3_O_4_ through the reduction of mesoporous *α*−Fe_2_O_3_. The process began with the utilization of ordered mesoporous *α*−Fe_2_O_3_, into which a solution containing Fe(NO_3_)_3_·9H_2_O, ethanol, and mesoporous silica (KIT−6) was absorbed to yield a dry powder. This powder was then subjected to heating at 600 °C in air for 6 h. Subsequent treatment with a hot 2 mol/L NaOH solution led to the formation of *α*−Fe_2_O_3_. Finally, reduction was achieved by subjecting the material to heating at 350 °C for 1 h under a 5%H_2_−95%Ar atmosphere, resulting in the transformation into Fe_3_O_4_. Almeida [29] investigated the thermal reduction of hydrothermally synthesized *α*−Fe_2_O_3_ nanorods to Fe_3_O_4_ nanorods under a hydrogen atmosphere. Initially, *α*−Fe_2_O_3_ nanorods were fabricated via the hydrothermal synthesis of an aqueous solution of FeCl_3_ in the presence of NH_4_H_2_PO_4_ surfactant at 200 °C for 2 h. Subsequently, subjecting the *α*−Fe_2_O_3_ nanorods to in situ heating at 360 °C under an H_2_ atmosphere with a pressure of 1 bar for 0.5 h facilitated their complete transition to Fe_3_O_4_. Furthermore, Tong [30] also synthesized urchin-like Fe_3_O_4_ nanostructures from the *α*−FeOOH precursor under a reducing atmosphere. Initially, *α*−FeOOH was obtained through a glucose-guided hydrolyzing approach by refluxing a mixture of glucose, FeSO_4_·7H_2_O, and deionized water at 60 °C for 12 h under magnetic stirring. The formation of the urchin-like Fe_3_O_4_ occurred at a temperature range of 350~400 °C for 2 h in 20%H_2_/80%Ar mixing gas with a flow rate of 1.0 L/min. Finally, the system was allowed to naturally cool down to room temperature under the protection of N_2_. Therefore, there is a need for a synthesis pathway for Fe_3_O_4_ that employs simple raw materials and operations with low energy consumption. In this work, we present a facile one-step solvothermal approach for the synthesis of highly pure and magnetically separable Fe_3_O_4_ photocatalyst with a distinctive flower-like morphology, which demonstrates remarkable efficiency in the complete removal of azo dye contaminants.

## 2. Materials and Methods

### 2.1. Materials

FeCl_3_ (99.9%), urea (99.999%), Methyl Orange (≥96%), Orange G (≥96%), and Acid Red 37 (with a strength of 200%) were obtained from Aladdin Co., Ltd. (Shanghai, China); methyl alcohol (MA; ≥99.5%), ethyl alcohol (EA; ≥99.7%), ethylene glycol (EG; ≥99.5%), and hydrogen peroxide (H_2_O_2_; 30 wt.%) were supplied by Chengdu Kelong Chemical Co., Ltd. (Chengdu, China). All reagents were used without additional purification. Purified water (H_2_O) served as the reaction medium or eluent in all experiments and was obtained from the HRO402 ultra-pure water device manufactured by Chengdu Zhonghan Water Treatment Equipment Co., Ltd. (Chengdu, China). Moreover, the general characteristics of Methyl Orange, Orange G, and Acid Red 37 dyes, including molecular weight, chemical structure, pictures, and Ultraviolet-Visible (UV−VIS) patterns of 10 mg/L dyes, and maximum absorption wavelengths (λ_max_), are shown in Table 1.

### 2.2. Synthesis

**Figure 1:**
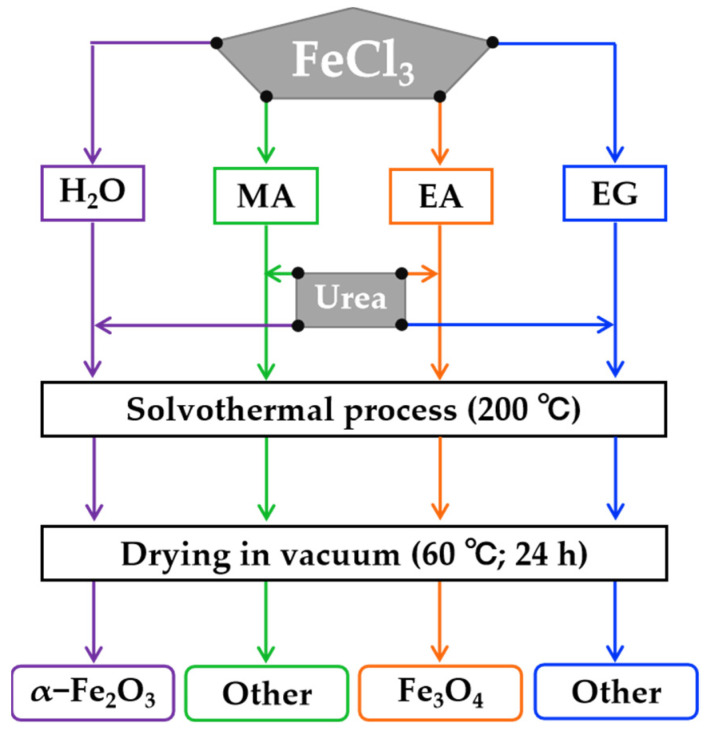
Schematic illustration of the synthesis of samples using different solvents (H_2_O, MA, EA, and EG).

A simple solvothermal method, which did not involve a subsequent roasting process in a H_2_ atmosphere, was employed to synthesize magnetic Fe_3_O_4_, as referenced in our previous report [31]. Moreover, a flow chart of the synthesis procedure of samples was employed, as shown in Figure 1. In brief, FeCl_3_ (10 mmol) and the desired amounts of urea (0, 20, 40, 60, 80, 100, 120, and 140 mmol) were dissolved into 60 mL of different solvents (MA, EA, EG, and H_2_O) with stirring for 1.5 h. Subsequently, the mixed solution was decanted and sealed into a hydrothermal synthesis reactor with a capacity of 100 mL and kept at a temperature of 200 °C for various durations (3, 6, 12, 24, 36, and 48 h). After the reaction, the resulting precipitates were collected and washed with H_2_O and EA, then dried under vacuum at 60 °C for 24 h.

### 2.3. Characterization

The phase compositions of the samples were identified by a DX-2700 X-ray diffractometer (XRD; Dandong Haoyuan Instrument Co., Ltd., Dandong, China). The morphology and size of samples were examined by an SEM-5000 scanning electron microscope (SEM; CIQTEK Co., Ltd., Hefei, China). The Fe2p XPS spectrum of the Fe_3_O_4_ sample was determined by X-ray photoelectron spectroscopy (XPS; ESCALAB 250Xi; Thermo Fisher Scientific, Waltham, MA, USA). The magnetic properties of the Fe_3_O_4_ sample were examined by a Lakeshore 7307 vibrating sample magnetometer (VSM; Novi, MI, USA).

### 2.4. Magnetic Response Behaviors of Samples

The resulting powder sample, weighing 0.1 g, was placed in a vial containing 30 mL of H_2_O. Subsequently, it underwent ultrasonic dispersion for 5 min and was allowed to stand for 6 h. Finally, a Nd−Fe−B magnet (Jinhua Yuanqiao Magnetic Industry Co., Ltd., Jinhua, China) was positioned on one side of the vial for a duration of 10 s. The dimensions of the vial were 27.5 mm in diameter and 72.5 mm in height; the Nd−Fe−B magnet measured 60.0 mm in diameter and 30.0 mm in height.

### 2.5. Photodegradation of Dyes

The catalytic activity of resulting Fe_3_O_4_ was evaluated by the photodegradation of various dyes (including Methyl Orange, Orange G, and Acid Red 37) under UV light illumination. Briefly, about 100 mg of Fe_3_O_4_ powders and 1.0 mL of H_2_O_2_ were dispersed into 100 mL of dye aqueous solution with a concentration of 10 mg/L. Later, the mixed solution was then illuminated by simulated UV light from a medical ultraviolet lamp (U = 220 V, P = 300 W, λ = 254 nm; Dancheng, China). At specified time intervals, about 10 mL of the suspension was extracted and promptly separated using a Nd−Fe−B magnet. Subsequently, the absorbance of the supernatant at the maximum absorption wavelength was measured utilizing a U-3900 UV−VIS spectrophotometer (Hitachi Ltd., Tokyo, Japan). Finally, the degradation rate (D (%)) of dye was calculated from the following equation: D (%) = (A_0_ − A_t_)/A_0_ × 100, where A_0_ represents the absorbance of the initial dye solution at a concentration of 10 mg/L and A_t_ represents the absorbance of the supernatant at a specific UV illumination time t. All experiments were conducted three times, and the average value was used for analysis.

### 2.6. Reuse of Fe_3_O_4_ Catalyst

After the first degradation and magnetic separation, the Fe_3_O_4_ particles were mixed with 100 mL of 10 mg/L dye solution and 1.0 mL of H_2_O_2_ and illuminated by UV light illumination for 2 h under continuous stirring, during which about 10 mL suspension was taken every 0.5 h to obtain the residual dye solution through magnetic separation. Subsequently, all the removed catalyst and dye solution were reintroduced into the reaction system after testing its absorbance.

## 3. Results and Discussion

**Figure 2:**
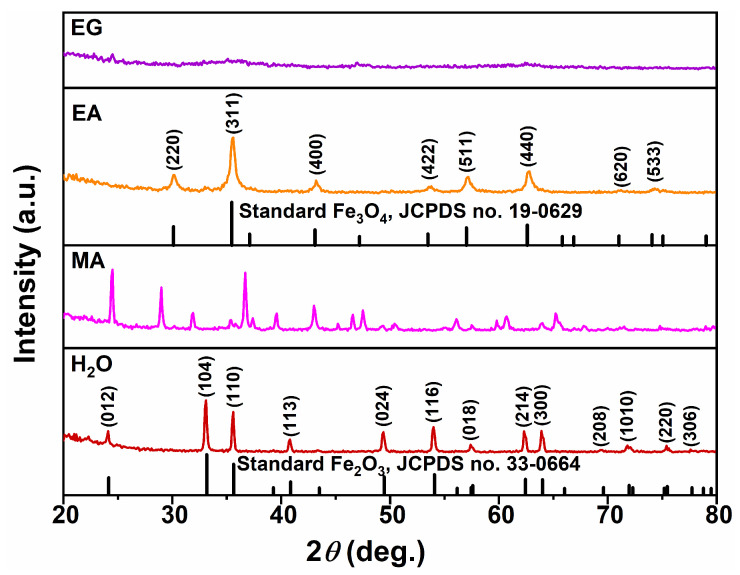
XRD patterns of the samples synthesized under different single-solvent systems of H_2_O, MA, EA, and EG at 200 °C for 12 h with a urea amount of 80 mmol.

Figure 2 illustrates the XRD patterns of samples synthesized under different single-solvent systems (H_2_O, MA, EA, and EG) at 200 °C for 12 h with a urea amount of 80 mmol. In the reaction media of the H_2_O and EA systems, the XRD patterns revealed distinct diffraction peaks consistent with standard hexagonal α−Fe_2_O_3_ (hematite, JCPDS no. 33-0664) and standard cubic Fe_3_O_4_ (magnetite, JCPDS no. 19-0629). The Miller indices corresponding to each diffraction peak were also indicated, as illustrated in Figure 2. This suggests that the predominant components of the resulting samples obtained in these two solvent systems were hexagonal α−Fe_2_O_3_ and cubic Fe_3_O_4_, respectively. In contrast, for the reaction medium of the MA system, numerous sharp diffraction peaks were observed in the XRD pattern, with no single phase matching any known standard from JCPDS. It can be inferred that this resulting sample was composed of at least two phases based on its chaotic diffraction peaks. Furthermore, when EG was used as the reaction system, a gentle curve was observed in the XRD pattern of the as-obtained sample, suggesting that it mainly consisted of an amorphous substance.

**Figure 3:**
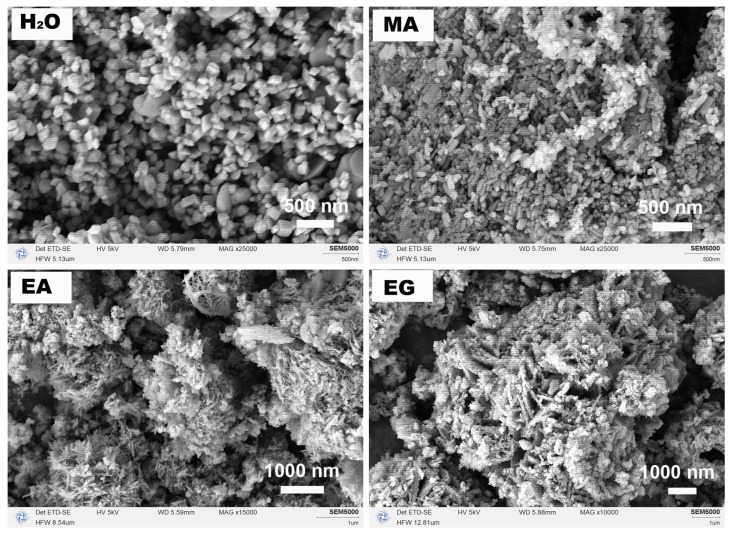
SEM images of the samples synthesized under different single-solvent systems of H_2_O, MA, EA, and EG at 200 °C for 12 h with a urea amount of 80 mmol.

Figure 3 shows the corresponding SEM images of samples synthesized under various single-solvent systems (H_2_O, MA, EA, and EG). The morphology of hexagonal α−Fe_2_O_3_ synthesized in an inorganic solvent (H_2_O) exhibited a predominantly polyhedral structure with an average size of approximately 100 nm. Conversely, when the reaction system utilized the organic solvent of MA, the resulting sample manifested as a large agglomeration comprised of nanometer rice granular particles. Upon changing the reaction system to employ EA as the organic solvent, the morphology of cubic Fe_3_O_4_ transformed into micron-scale aggregates formed by interweaving nanoneedles. Furthermore, when EG was utilized as the reaction medium, amorphous substances took on a primarily 3D flower-like microsphere form through interactions with nanoplate structures, and some micron-scale small particles were observed to be attached to them. Based on both XRD and SEM analysis results aforementioned in Figure 1, it can be inferred that the nature of the reaction medium not only had a significant impact on the composition of the product phase but also exerted a great influence on the morphology of the product. Given the well-known magnetic sensitivity of Fe_3_O_4_, our next step involves exploring EA as a potential solvent for further experimentation.

**Figure 4:**
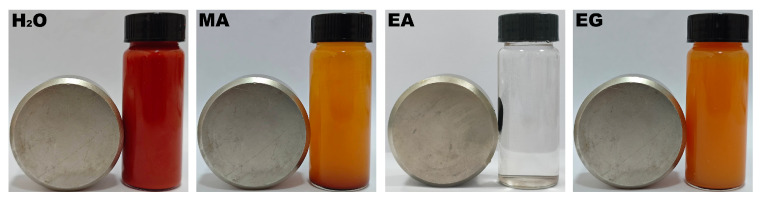
Magnetic response behaviors of the samples synthesized under different single-solvent systems of H_2_O, MA, EA, and EG at 200 °C for 12 h with a urea amount of 80 mmol.

Figure 4 shows the magnetic response behaviors of the samples synthesized under different single-solvent systems of H_2_O, MA, EA, and EG at 200 °C for 12 h with a urea amount of 80 mmol. As illustrated in Figure 4, only the product synthesized in the solvothermal system utilizing EA as the reaction medium demonstrated magnetically sensitive properties. The XRD analysis presented in Figure 1 indicates that its phase was identified as cubic Fe_3_O_4_. The black particles dispersed in the aqueous solution could be attracted to the side of the vial within 10 s by a Nd−Fe−B magnet, resulting in a clear and transparent aqueous solution. Consequently, subsequent research can concentrate on the reaction system employing EA as the reaction medium.

**Figure 5:**
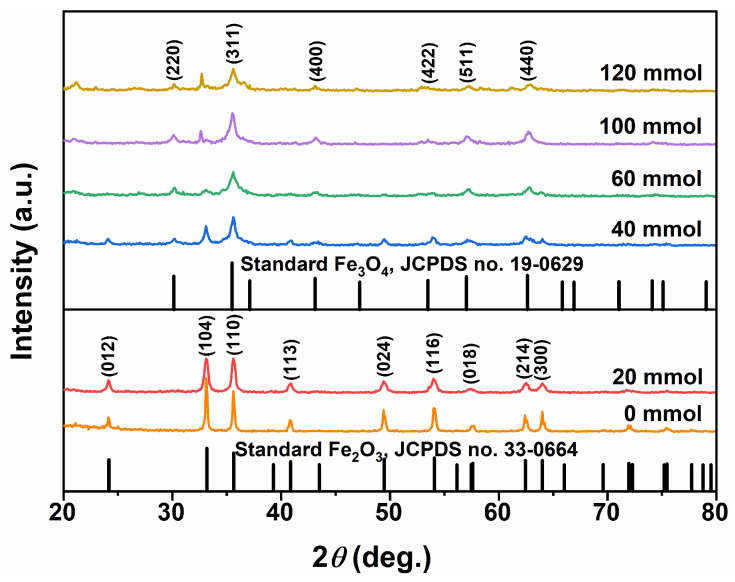
XRD patterns of the samples synthesized in an organic solvent system using EA at 200 °C for 12 h with varying urea amounts of 0, 20, 40, 60, 100, and 120 mmol.

By utilizing EA as the reaction medium in the solvothermal process, the impact of urea addition on the phase composition and morphology of the resulting product was investigated. Figure 5 depicts the XRD patterns of the samples obtained from a gradient experiment involving urea addition. When no urea was added (i.e., 0 mmol), the XRD pattern of the resulting product matched that of standard hexagonal α−Fe_2_O_3_ (hematite, JCPDS no. 33-0664), indicating a single component product. With an addition of 20 mmol urea, although still displaying characteristic diffraction peaks of single-phase α−Fe_2_O_3_, there was a slight reduction in diffraction intensity compared to the α−Fe_2_O_3_ sample obtained without urea added. Upon increasing the urea addition to 40 mmol, a weak diffraction peak of cubic Fe_3_O_4_ was detected alongside characteristic diffraction peaks of α−Fe_2_O_3_. A further increase to 60 mmol resulted in dominant characteristic diffraction peaks of Fe_3_O_4_ with very weak characteristic diffraction peaks of α−Fe_2_O_3_, indicating that Fe_3_O_4_ became the main component. Subsequent increases in urea dosage to 100 mmol and 120 mmol led to samples consisting of mixtures containing both Fe_3_O_4_ and α−Fe_2_O_3_ phases.

**Figure 6:**
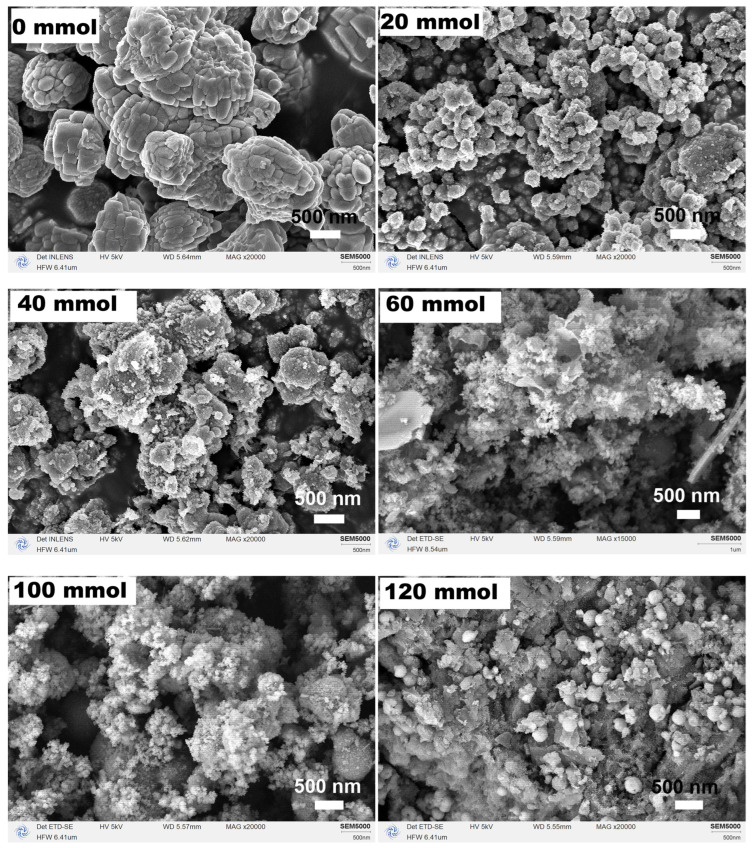
SEM images of the samples synthesized in an organic solvent system using EA at 200 °C for 12 h with varying urea amounts of 0, 20, 40, 60, 100, and 120 mmol.

Figure 6 depicts the corresponding SEM images of the products. The morphology of α−Fe_2_O_3_ synthesized without the addition of urea consisted of an elliptic aggregate formed by the directional self-assembly of numerous small particles with long axes measuring 600 nm. Upon the addition of 20 mmol urea, the size of α−Fe_2_O_3_ particles decreased, with a single particle measuring approximately 300 nm, while the particle size in the sample with 40 mmol urea exhibited differences. As the amount of urea was increased to 60 mmol, the grainy particles disappeared and were replaced by wrinkled shredded paper-like structures, accompanied by a large number of bumps on their surfaces, which transformed into needle-like substances as the urea addition reached 80 mmol (see Figure 3). Subsequently, increasing the amount of urea to 100 mmol and then to 120 mmol resulted in the disappearance of the needles and their replacement by particles of varying sizes.

**Figure 7:**
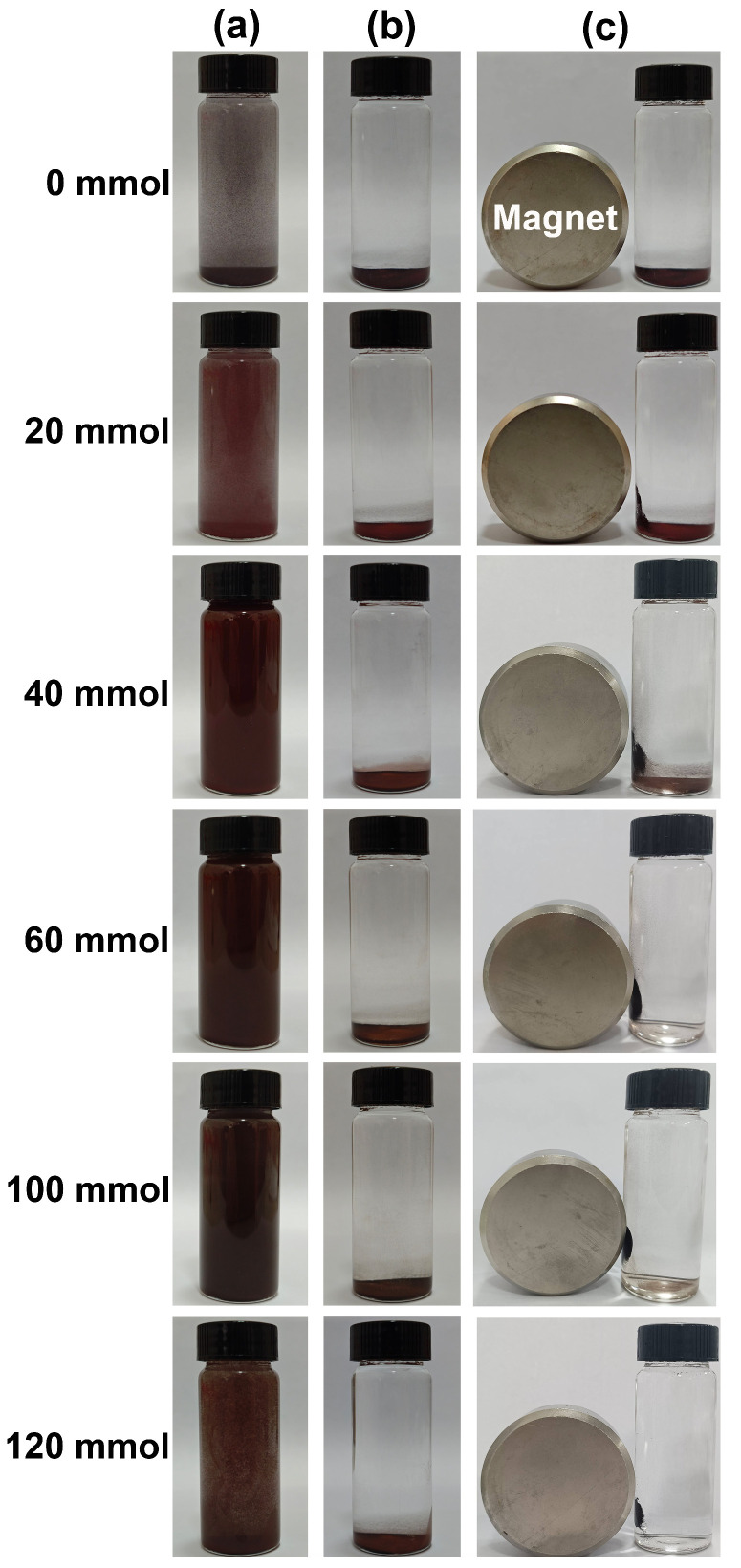
Magnetic response behaviors of the samples synthesized in an organic solvent system using EA at 200 °C for 12 h with varying urea amounts of 0, 20, 40, 60, 100, and 120 mmol. (**a**) A powder sample weighing 0.1 g was dispersed in a solution of 30 mL H_2_O using ultrasound for a duration of 5 min. (**b**) The mixture was then allowed to stand for 6 h. (**c**) Finally, a Nd−Fe−B magnet was placed on one side of the vial for 10 s.

Figure 7 shows the magnetic response behaviors of the samples synthesized in an organic solvent system using EA at 200 °C for 12 h with varying urea amounts of 0, 20, 40, 60, 80, 100, and 120 mmol. As illustrated in Figure 7a, the sedimentation of particles in water gradually diminished with increasing urea addition, leading to improved dispersion. After standing for 6 h, all particles settled at the bottom of the vial, resulting in a clarified and transparent upper layer (as shown in Figure 7b). Subsequently, a Nd−Fe−B magnet was positioned on the left side of the vial, as shown in Figure 7c. It was observed that when no urea was added, the particles at the bottom of the vial did not respond to the magnetic field. However, some particles began to be attracted toward the magnet’s side. When the urea concentration increased to 60 mmol, all particles at the bottom were drawn entirely toward that side of the vial by magnetic force. The results presented in Figure 7 indicate that when urea content exceeded 60 mmol, the resulting powder sample exhibited magnetically sensitive characteristics, thus enabling rapid solid−liquid separation under magnetic influence.

**Figure 8:**
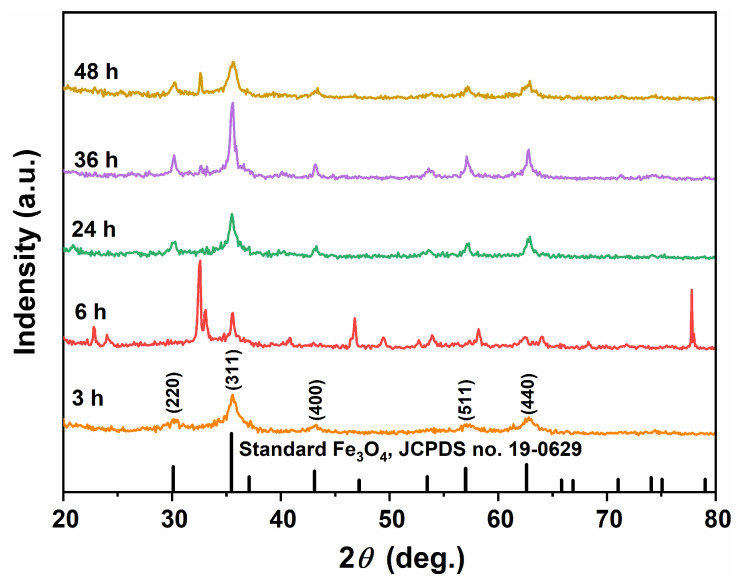
XRD patterns of the samples synthesized in an organic solvent system using EA at 200 °C for varying durations (3, 6, 24, 36, and 48 h) with a urea amount of 80 mmol.

Combined with the above XRD phase composition analysis and SEM morphology analysis, it was determined that the product obtained through a solvothermal reaction at 200 °C for 12 h using EA as the reaction medium and adding 80 mmol of urea was pure Fe_3_O_4_, exhibiting a unique acicular interwoven aggregate morphology. Subsequently, the impact of solvothermal reaction time on the phase composition and morphology of the product was further investigated. Figure 8 presents the XRD patterns of the products obtained at 200 °C after varying solvothermal reaction times with EA as the reaction medium and 80 mmol of urea added. The XRD pattern of the sample obtained after a solvothermal reaction time of 3 h displayed four faint diffraction peaks consistent with standard cubic Fe_3_O_4_ (magnetite, JCPDS no.19-0629), indicating incomplete crystallization and poor crystallinity. As the reaction time extended to 6 h, 36 h, and 48 h, additional distinguished diffraction peaks appeared alongside those characteristic of cubic Fe_3_O_4_, suggesting that these samples contained other phases besides Fe_3_O_4_ and were not single-phase compositions. Notably, when the solvothermal reaction time reached 24 h, the XRD diffraction peaks corresponding to heterophase were relatively weak compared to that of the cubic Fe_3_O_4_ phase, indicating that cubic Fe_3_O_4_ was its main component. The results from XRD analysis in Figure 8 contrasted sharply with those in Figure 2; they provided valuable insights into process parameters for obtaining pure Fe_3_O_4_ with complete crystallinity, utilizing a reaction medium consisting of 60 mL EA along with an addition of 80 mmol urea during a solvothermal reaction lasting for 12 h at 200 °C.

**Figure 9:**
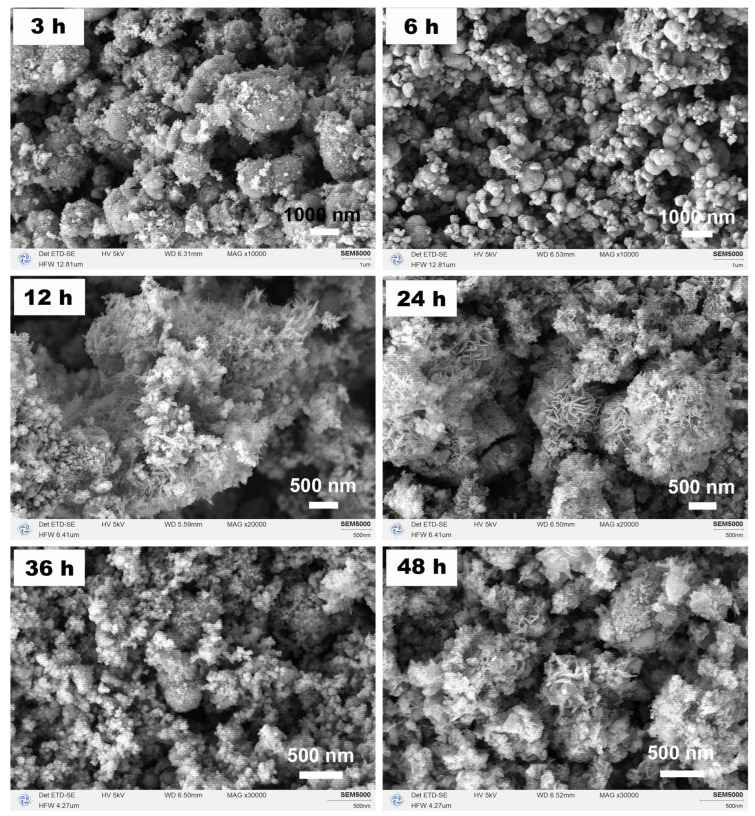
SEM images of the samples synthesized in an organic solvent system using EA at 200 °C for varying durations (3, 6, 12, 24, 36, and 48 h) with a urea amount of 80 mmol.

Figure 9 depicts the corresponding SEM images, including the solvothermal reaction for 12 h, which was a higher magnification SEM image of the sample shown in Figure 3. As the solvothermal reaction time increased, there was an intriguing evolution in the morphology of the sample: from globular particles of varying sizes to acicular interwoven aggregates, then to flaky interwoven aggregates, and finally to crumpled scraps of paper. When combined with the XRD analyses in Figure 8, it can be inferred that, initially, Fe_3_O_4_ with low crystallinity was obtained during the continuous solvothermal reaction process, followed by the appearance of other phases. Subsequently, the heterogeneous phase disappeared, and pure Fe_3_O_4_ with high crystallinity formed before the reemergence of heterogeneous phases occurred. During this period, it was likely that dissolution and recrystallization processes continued while self-assembly occurred under the influence of EA and its derivatives resulting from reactions within the solvothermal closed system, ultimately leading to continuous evolution in morphology.

**Figure 10:**
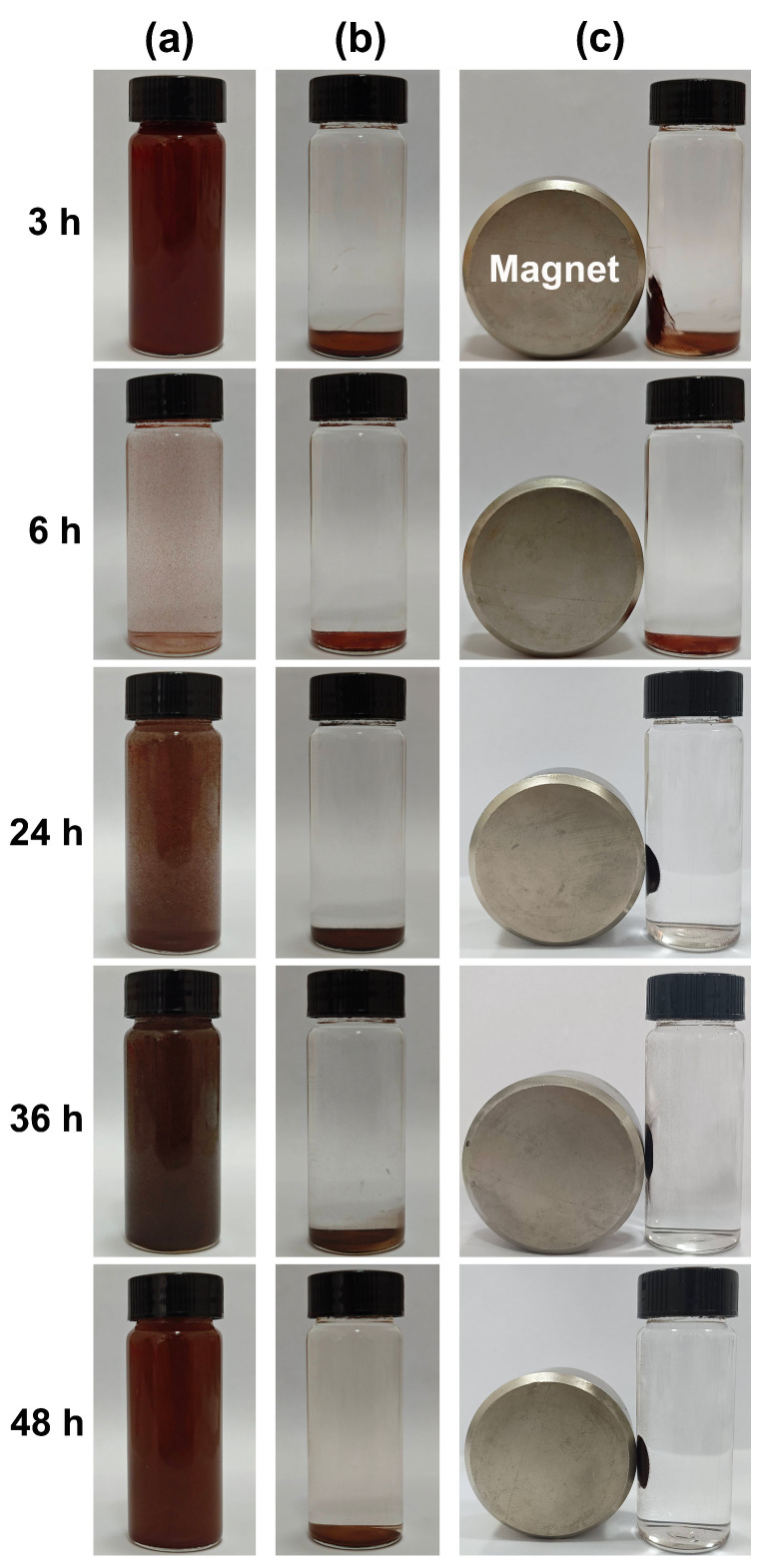
Magnetic response behaviors of the samples synthesized in an organic solvent system using EA at 200 °C for varying durations (3, 6, 24, 36, and 48 h) with a urea amount of 80 mmol: (**a**) A powder sample weighing 0.1 g was dispersed in a solution of 30 mL H_2_O using ultrasound for a duration of 5 min. (**b**) The mixture was then allowed to stand for 6 h. (**c**) Finally, a Nd−Fe−B magnet was placed on one side of the vial for 10 s.

Figure 10 shows the magnetic response behaviors of the samples synthesized in an organic solvent system using EA at 200 °C for varying durations of 3, 6, 24, 36, and 48 h with a urea amount of 80 mmol. As illustrated in Figure 10a, the sample obtained after 6 h of the solvothermal reaction exhibited a more pronounced sedimentation phenomenon, followed by the sample synthesized at 12 h. In contrast, other samples demonstrated good dispersion in H_2_O. After standing for 6 h, as depicted in Figure 10b, all particles within the vials settled at the bottom, resulting in a clear and transparent upper layer. Subsequently, a Nd−Fe−B magnet was positioned on the left side of the vial, as shown in Figure 10c. At this point (3 h into the solvothermal reaction), only a portion of the particles was attracted to that side of the vial; however, no attraction was observed at 6 h. When considering Figure 7 alongside these observations, it became evident that samples obtained after 12 h of solvothermal reaction exhibited excellent magnetic sensitivity characteristics and could be rapidly attracted to the side of the vial when exposed to magnets.

**Figure 11:**
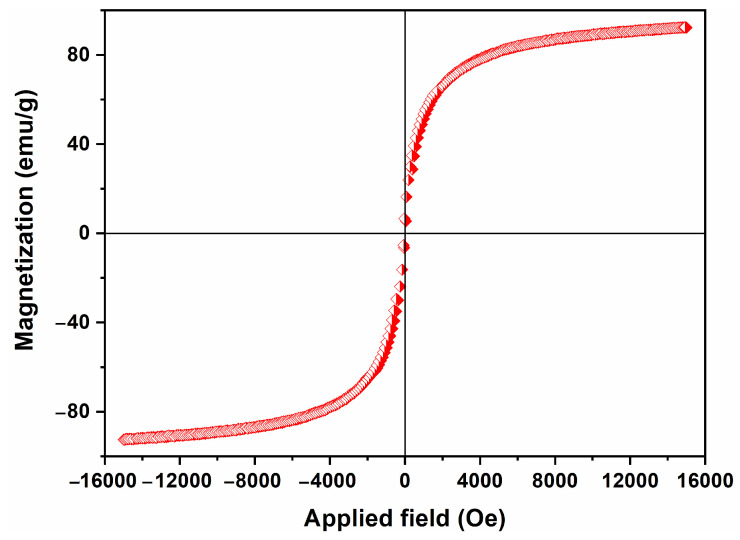
A magnetic hysteresis loop of Fe_3_O_4_ synthesized in an organic solvent system using EA at 200 °C for 12 h with a urea amount of 80 mmol.

The magnetic hysteresis loop of the Fe_3_O_4_ synthesized in an organic solvent system using EA at 200 °C for 12 h with a urea amount of 80 mmol was measured using a VSM at room temperature. The applied magnetic field ranged from −15,000 to 15,000 Oe. As shown in Figure 11, the hysteresis loops nearly overlap, and they pass through the origin of the axes, indicating an absence of retentivity and coercivity. This observation suggests that the resulting Fe_3_O_4_ can be classified as a soft magnetic material exhibiting superparamagnetic properties. Furthermore, it was observed that the magnetization reached 92.2 emu/g at 15,000 Oe. This value is not only comparable to but also may even surpass those reported in other studies [32,33]. Such a finding highlights its exceptional magnetic sensitivity and serves as the foundation for its distinctive magnetic response behavior.

**Figure 12:**
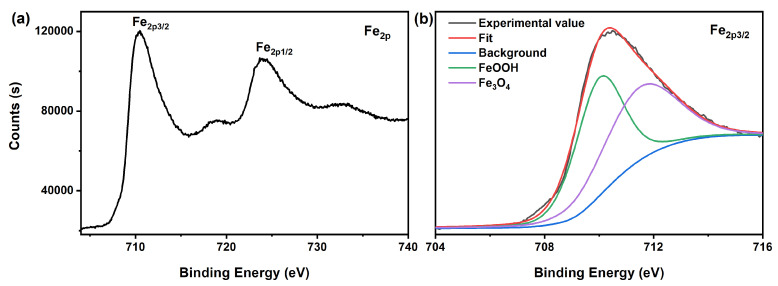
(**a**) Fe2p core-levels XPS spectrum and (**b**) the Fe_2p3/2_ peak in different chemical environments of Fe_3_O_4_ synthesized in an organic solvent system using EA at 200 °C for 12 h with a urea amount of 80 mmol.

The surface properties of magnetic Fe_3_O_4_ synthesized in an organic solvent system using EA at 200 °C for 12 h with a urea amount of 80 mmol were investigated through XPS. As shown in Figure 12a, the peaks corresponding to Fe_2p3/2_ and Fe_2p1/2_ in Fe_3_O_4_ were observed at binding energies of 710.4 eV and 724.1 eV, respectively. This indicates the presence of nonequivalent Fe components existing within different chemical environments in the Fe_3_O_4_ samples. Furthermore, the Fe_2p3/2_ peak at 710.4 eV can be accurately characterized according to data from the NIST XPS Database (Version 4.1) as well as relevant literature [34], as illustrated in Figure 12b. These findings provide clear evidence for the presence of the Fe_3_O_4_ phase within the magnetic sample, which is consistent with previous results obtained from XRD in Figure 2.

**Figure 13:**
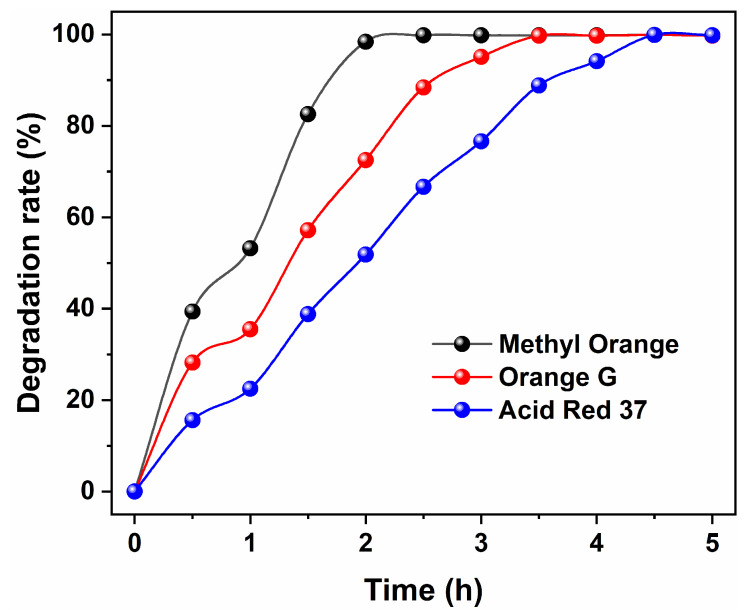
Photocatalytic degradation of Methyl Orange, Orange G, and Acid Red 37 dyes upon magnetic Fe_3_O_4_ catalyst synthesized in an organic solvent system using EA at 200 °C for 12 h with a urea amount of 80 mmol.

Based on the above analyses, it is evident that Fe_3_O_4_ with a unique morphology, complete crystallization, and excellent magnetic sensitivity can be obtained by utilizing EA as a solvent and adding 80 mmol urea at 200 °C for a solvothermal reaction lasting 12 h. Subsequently, the resulting magnetic Fe_3_O_4_ under these conditions was employed as a photocatalyst to assess its degradation effect on three azo dyes, including Methyl Orange, Orange G, and Acid Red 37. Figure 13 illustrates the curves depicting the photocatalytic degradation rate of the as-obtained Fe_3_O_4_ for Methyl Orange, Orange G, and Acid Red 37 dyes over time. As observed in Figure 13, the degradation rate of all three azo dyes was relatively rapid during the initial 0.5 h of the photocatalytic degradation reaction. This phenomenon might be attributed to both physical adsorption and photocatalysis occurring simultaneously onto Fe_3_O_4_. As UV light continued to irradiate, the degradation of all three dyes persisted. Almost complete degradation (D > 99%) of Methyl Orange dye was achieved after a photocatalytic reaction lasting for 2.0 h, whereas Orange G and Acid Red 37 dyes reached similar levels after reactions lasting for 3.5 and 4.5 h, respectively. It was noteworthy that Fe_3_O_4_ exhibited significantly better photocatalytic degradation effects on Methyl Orange compared to those observed with respect to Orange G and Acid Red 37. This difference in performance might be attributed to Methyl Orange having a smaller relative molecular weight compared to the other two dyes despite being an azo dye. Consequently, we selected Methyl Orange dye as our target for further investigation into the reuse performance of Fe_3_O_4_ catalysts.

**Figure 14:**
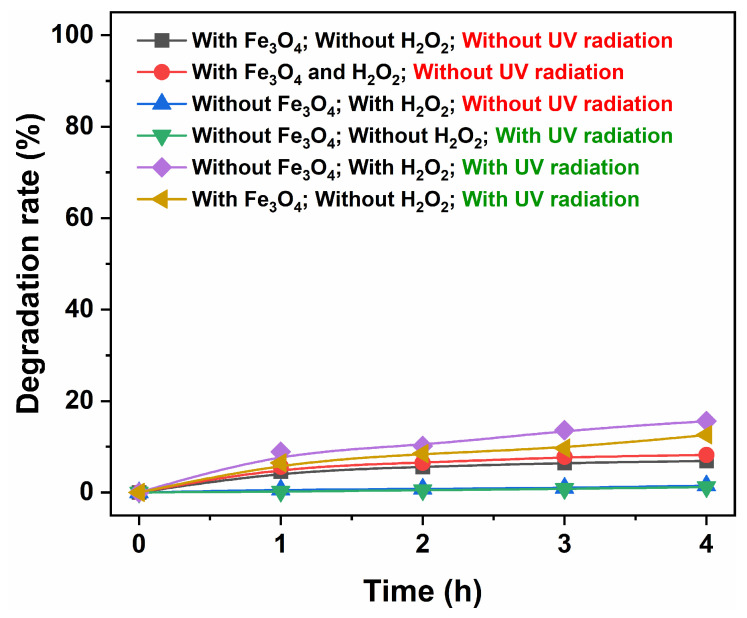
Controlled experiments investigating the degradation of Methyl Orange. (The magnetic Fe_3_O_4_ catalyst was synthesized in an organic solvent system using EA at 200 °C for 12 h with a urea amount of 80 mmol.).

Figure 14 presents six controlled experiments investigating the degradation of Methyl Orange. In the scenario where only Fe_3_O_4_ catalyst was used, without H_2_O_2_ and UV radiation, the degradation rate of Methyl Orange after 4 h was merely 6.9%. This indicates that the dark adsorption effect of Fe_3_O_4_ on Methyl Orange was minimal. When both the Fe_3_O_4_ catalyst and H_2_O_2_ were present without UV radiation, the degradation rate at 4 h increased to 8.2%. Conversely, in the presence of only H_2_O_2_, the degradation rate remained low at just 1.5%. These results suggest that while H_2_O_2_ exhibits a limited capacity for degrading Methyl Orange, UV radiation plays a crucial role in enhancing the catalytic activity of Fe_3_O_4_. In an experiment involving solely UV radiation, the degradation rate of Methyl Orange over a period of 4 h was found to be only 1.2%. However, under UV radiation exposure, H_2_O_2_ managed to degrade up to 15.6% of Methyl Orange, whereas Fe_3_O_4_ catalyst achieved a degradation rate of 12.6% for Methyl Orange when subjected to UV radiation. The findings indicate that both Fe_3_O_4_ and H_2_O_2_ exhibit some degree of effectiveness in degrading Methyl Orange under UV irradiation; however, this effect remains relatively weak. These controlled experiments further corroborate the mechanism underlying photo-Fenton-type reaction, as in our previous reports [31,35].

**Figure 15:**
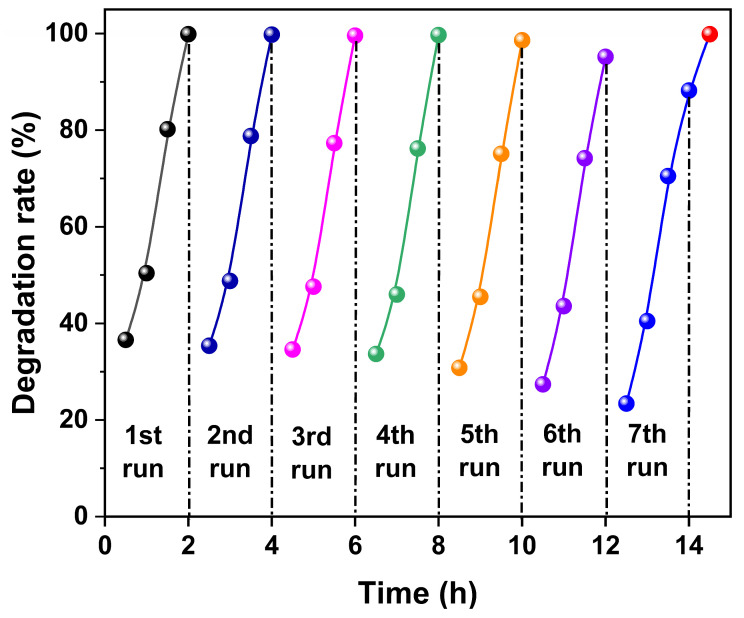
Regeneration of Methyl Orange dye in the presence of magnetic Fe_3_O_4_ catalyst synthesized in an organic solvent system using EA at 200 °C for 12 h with a urea amount of 80 mmol.

Figure 15 illustrates the regeneration of Methyl Orange dye magnetic Fe_3_O_4_ catalyst synthesized in an organic solvent system using EA at 200 °C for 12 h with a urea amount of 80 mmol. In the first five cycles of reusing the Fe_3_O_4_ catalyst, the almost complete degradation (D > 99%) of Methyl Orange dye was achieved. However, in the sixth cycle, some residue of Methyl Orange dye was observed in the solution, but the degradation rate still reached 95.2%, and the degradation rate for the seventh cycle was 88.2%. It is noteworthy that an additional 0.5 h of UV irradiation after the end of the seventh cycle could completely degrade the residual Methyl Orange dye in the solution. The excellent photocatalytic activity, proven regeneration performance, and outstanding magnetic sensitivity of Fe_3_O_4_ provide a strong basis for its potential as a photocatalyst candidate for the degradation of azo dyes.

Combined with the XRD and SEM analysis, it can be inferred that a subtle synergistic effect existed between the reaction medium and urea in the solvothermal process, jointly determining the crystal structure, composition, and morphology of the final product. Additionally, both the amount of urea added and the solvothermal reaction time influenced the final form of the product. The solvothermal reaction involved three main processes: First, solvent molecules reacted to generate water; second, some urea underwent hydrolysis to produce NH_3_ gas and CO_2_ gas. This not only increased pressure within the system but also made it alkaline because of dissolved NH_3_, promoting crystallization. Finally, under high-temperature and -pressure conditions in a closed solvent thermal system, urea could condense into biuret, triuret, and cyanic acid. Biuret and cyanic acid are reductive agents that facilitate Fe^3+^ reduction to Fe^2+^. These interacting processes ultimately yield Fe_3_O_4_ or α−Fe_2_O_3_, along with their compounds/complexes. Variations in crystal structure and composition affected magnetic sensitivity properties, which subsequently influenced photocatalytic activity.

## 4. Conclusions

A one-step solvothermal synthesis strategy for magnetic Fe_3_O_4_ was proposed, utilizing solvent and urea as inducing agents. The optimal process parameters for synthesizing single-phase Fe_3_O_4_ were determined by incorporating urea and adjusting solvothermal time: 10 mmol of FeCl_3_, 80 mmol of urea, and 60 mL of EA were used as dosages, with a solvothermal process of 200 °C for 12 h, followed by drying at 60 °C for 24 h under vacuum. The resulting Fe_3_O_4_ was a cubic magnetite characterized by excellent crystallinity and acicular agglomerate morphology. More than 99% degradation was achieved after photocatalytic reactions lasting for 2.0 h (Methyl Orange), 3.5 h (Orange G), and 4.5 h (Acid Red) in the presence of the as-obtained Fe_3_O_4_. When specifically focusing on the degradation of Methyl Orange dye, it was observed that during the initial five cycles of repeated photocatalysis, Fe_3_O_4_ was able to achieve complete degradation within 2.0 h. However, by the end of the seventh cycle, the degradation rate decreased to 88.2%. Nevertheless, extending the UV irradiation by an additional 0.5 h could lead to complete degradation of the remaining Methyl Orange dye. Furthermore, the Fe_3_O_4_ exhibited excellent magnetically sensitive characteristics and had been demonstrated to be superparamagnetic, with a magnetization of 92.2 emu/g, allowing catalyst particles to be quickly recovered in heterogeneous systems, which reduced recovery costs, shortened recovery cycles, and minimized secondary pollution.

## Figures and Tables

**Table 1:** General characteristics of Methyl Orange, Orange G, and Acid Red 37 dye.

Dye Name	Molecular Weight	Chemical Structure	Picture of Dye (10 mg/L)	UV−VIS Pattern (10 mg/L)	λ_max_ (nm)
Methyl Orange	327.3	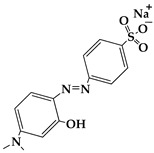	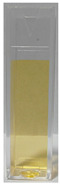	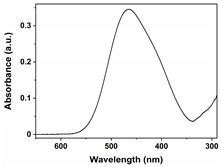	466
Orange G	452.4	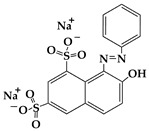	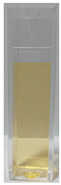	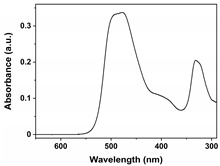	477
Acid Red 37	524.4	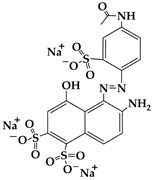	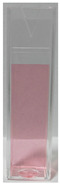	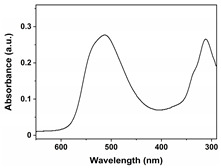	512

## Data Availability

Data are contained within the article.
